# Analysis of Metabolomic Changes in Xylem and Phloem Sap of Cucumber under Phosphorus Stresses

**DOI:** 10.3390/metabo12040361

**Published:** 2022-04-18

**Authors:** Jingjing Sun, Qinglin Li, Hui Xu, Wentao Zhang

**Affiliations:** School of Agricultural Engineering, Jiangsu University, Zhenjiang 212013, China; 2222016050@stmail.ujs.edu.cn (J.S.); 2221916039@stmail.ujs.edu.cn (H.X.); 2211916040@stmail.ujs.edu.cn (W.Z.)

**Keywords:** cucumber, phosphorus stress, xylem, phloem, metabonomics, GC-MS

## Abstract

Cucumber xylem and phloem sap is a key link in nutrient distribution, transportation and signal transduction of cucumber plants; however, the metabolic response mechanism of cucumber xylem and phloem sap under phosphorus stress has not been clearly revealed. In this study, gas chromatography-mass spectrometry (GC-MS) combined with principal component analysis (PCA) and partial least squares discriminant analysis (PLS-DA) were used to analyze the metabolites in cucumber xylem and phloem sap under different phosphorus stress. A total of 22 differential metabolites were screened from xylem and phloem sap, respectively. Through the analysis of metabolic pathways of differential metabolites, four and three key metabolic pathways were screened, respectively. The results showed that compared with the normal phosphorus level, the content of most amino acids in the key metabolic pathway increased in xylem but decreased in phloem both under low and high phosphorus stress levels. The contents of sucrose and glucose in phloem glycolysis pathway showed a positive correlation with the change of phosphorus nutrient levels. The tricarboxylic acid cycle was promoted in xylem and phloem of cucumber under low and high phosphorus nutrient levels, and the contents of malic acid and citric acid increased significantly. This study provided abundant biochemical information for the metabolic response and regulation strategies of cucumber xylem and phloem under phosphorus stress, and is committed to looking for more sensitive markers to evaluate the supply level of phosphorus nutrients in cucumber.

## 1. Introduction

Phosphorus, as one of a large number of nutrient elements necessary for plant growth and development [1], participates in plant metabolic processes in various forms, such as carbohydrate synthesis, nitrogen metabolism, carbohydrate synthesis and fat metabolism. Phosphorus also has an important impact on plant yield, quality and stress resistance [2,3], and the phosphorus nutrient status in plants plays a key role in plant growth regulation. In the early stage, the diagnosis of plant phosphorus nutrient status was mainly carried out by observing the macro characteristics of plants, such as the difference of height, length and color, as well as the difference of leaf surface roughness and texture characteristics [4]. However, before the external characteristics of plants change, in order to resist the harm caused by phosphorus stress, plants use their metabolic ability to produce a large number of small molecular compounds and restructure their metabolic network to maintain necessary metabolism. Therefore, at the molecular level, the method of detecting metabolic biomarkers can quickly and efficiently reflect whether the phosphorus nutrient supply level of plants is normal, and confirm the nutritional status of plants as soon as possible when the nutrient deficiency can still be remedied [5], and guide the reasonable fertilization scheme, which has great practical significance.

In recent years, the development of metabonomics technology has provided new ideas for the study of plant metabolism. Metabonomics mainly focuses on the study of small molecular compounds and related dynamic changes in plants, and the detection of a variety of metabolites in key metabolic pathways. It has been widely used to explore the resistance mechanism of plants under different stresses. Much research has been carried out on the changes of small molecular compounds in rice, wheat, tomato and other plants to abiotic stress, such as temperature, alkali, molybdenum and phosphorus [6,7,8,9]. As for phosphorus stress, Duan et al. studied the characteristics of root exudates release system of typical plants in plateau lakeside wetland under phosphorus stress based on GC-MS, and the results showed that the relative contents of phthalic acid, benzene dicarbonic acid and cyclohexanone in root exudates increased first and then decreased with the change of phosphorus concentration [10]. Isiaka et al. analyzed metabolites in oil palm leaves and roots after phosphate treatment for two weeks. Increased maltose level indicated active degradation of starch in roots. High levels of glycerol and stearate indicated that roots and leaves provided energy for cells through lipid storage in the absence of phosphorus [11]. Xie et al. analyzed the metabolites and gene expression characteristics of Dioscorea zingiberensis under low phosphorus stress by GC-MS derivatization and RNA-SEQ, and investigated the close relationship between differential metabolites and gene expression characteristics [12]. Ding et al. used ICP-OES, GC-MS and LC-MS to analyze the ionomics and metabolomics in tea plants under phosphorus stress. The results implied that the phosphorus stress severely disturbed the metabolism of minerals and metabolites in tea plants, which influenced the yield and quality of tea [13]. Gao et al. compared the effects of low nitrogen and phosphorus deficiencies stresses on various metabolites in lettuce leaves based on metabolomics technology, and preliminarily investigated the changes of potential metabolic biomarkers under low nitrogen and phosphorus stresses [5].

From the above research, it can be seen that metabonomics technology has been tried and applied in different plants to study the metabolic process and response mechanism of molecular compounds. Cucumber is one of the vegetables widely cultivated in China. Its nutritional level and growth status are also affected by phosphorus stress. Cucumber xylem and phloem sap, as the key elements in nutrient distribution, transportation and signal transmission of cucumber plants, contain rich materials and information [14]. Xylem sap contains amino acids, organic acids, plant hormones, proteins and so on. Its main function is to transport the water and mineral nutrients absorbed by cucumber roots from the soil to the aboveground part of cucumber plants [15]; phloem sap is mainly composed of sugars, amino acids, proteins, vitamins, plant hormones and inorganic salts. Its main function is to transport the photosynthetic products formed in cucumber leaves to each part of cucumber plant [16], but there are few studies on the metabolic changes of cucumber xylem and phloem sap under phosphorus stress at this stage. Therefore, in order to further explore the effects of phosphorus stress on the metabolites in cucumber xylem sap and phloem sap during growth, in this research, cucumber xylem sap and phloem sap were used as experimental samples to be analyzed. Based on gas chromatography-mass spectrometry (GC-MS) technology, the variation law of metabolites in cucumber xylem and phloem sap under different phosphorus levels were analyzed, and the response mechanism was explored. The metabolomics method was expected to find more sensitive and active markers under different phosphorus levels, so as to evaluate the phosphorus supply status of cucumber.

## 2. Results

### 2.1. Identification of Metabolites

Using ribitol as internal standard, the relative content of metabolites was calculated by internal standard standardization method. A total of 81, 76, 81, 88 and 78 metabolites were detected in xylem sap at different phosphorus levels (P25%, P50%, P75%, P100% and P150%). A total of 115, 115, 114, 121 and 118 metabolites were finally detected in phloem sap under different phosphorus levels (P25%, P50%, P75%, P100% and P150%). Three main compounds were found in both metabolites, namely amino acids, organic acids and sugars. The types and contents of sugars and amino acids detected in phloem sap were higher than those in xylem sap, which was consistent with the functional characteristics of xylem and phloem.

### 2.2. Principal Component Analysis (PCA) of Metabolites in Xylem and Phloem Sap of Cucumber under Phosphorus Stress

**Figure 1:**
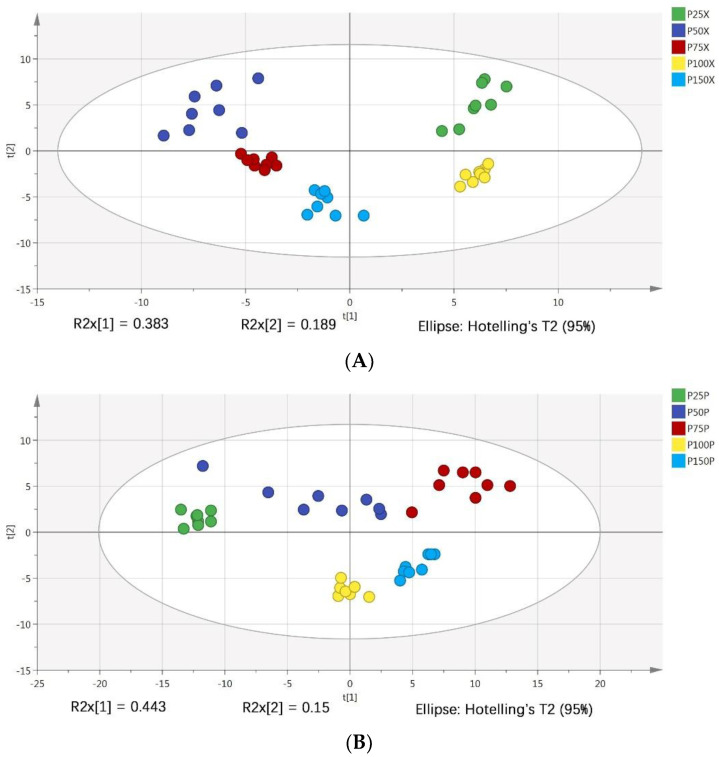
The PCA score maps of xylem (**A**) and phloem (**B**) sap.

PCA analysis was used to analyze the differences of cucumber xylem and phloem sap under five different phosphorus levels, which generally reflected the overall differences between the samples in each group and the degree of variation between the samples in the group. As shown in Figure 1A,B, the parallel samples of five groups of xylem and the parallel samples of five groups of phloem were both clustered together, indicating that all tests had good analytical stability and experimental reproducibility; at the same time, the interpretation rates of the first principal component and the second principal component of xylem were 38.3% and 18.9% respectively, and their cumulative contribution rates reached 57.2%. The interpretation rates of the first principal component and the second principal component of phloem were 44.3% and 15.0% respectively, and their cumulative contribution rates reached 59.3%. It can be seen that the xylem and phloem sap of cucumber both have an obvious separation trend under five phosphorus levels, which reflected the metabolite differences between these samples.

### 2.3. PLS-DA Analysis of Metabolites in Xylem and Phloem Sap of Cucumber under Phosphorus Stress

**Figure 2:**
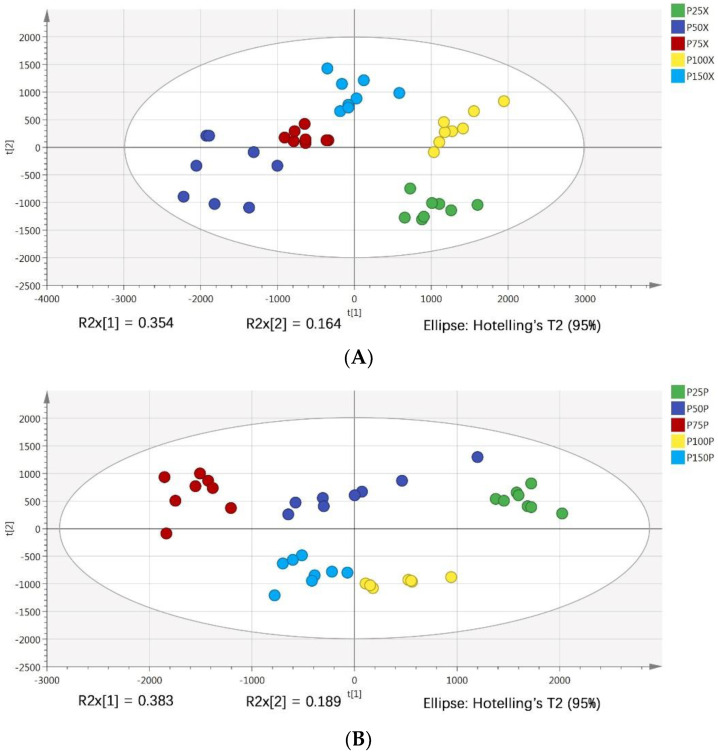

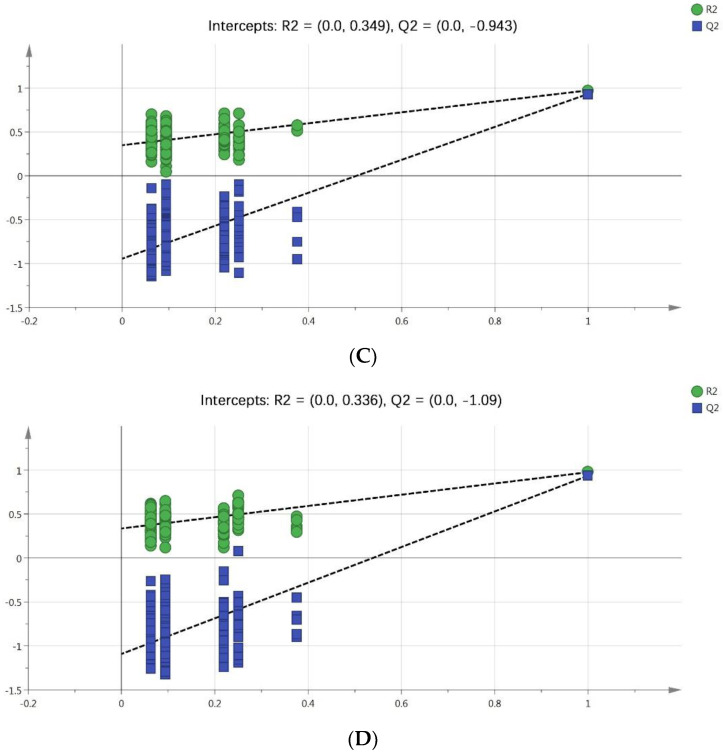
PLS-DA score maps of xylem and phloem sap (xylem (**A**), phloem (**B**)), the results of 200 randomly repeated tests (xylem (**C**), phloem (**D**)).

PLS-DA is a discriminant analysis method based on the classical partial least squares regression model. By properly rotating the principal components, PLS-DA can effectively distinguish the observed values between groups and find the influencing variables that lead to the difference between groups [17]. PLS-DA analysis was further used to verify the constructed PCA model. PLS-DA scores of cucumber xylem and phloem sap samples under five phosphorus levels were shown in Figure 2A,B. The results were similar to PCA. The cumulative discriminant interpretation ability of cucumber xylem sap of the model under positive ion mode was R^2^X = 0.849, R^2^Y = 0.97, and the prediction ability of the model was Q^2^ = 0.924, the cumulative discrimination and interpretation ability of cucumber phloem sap of the model under positive ion mode was R^2^X = 0.92, R^2^Y = 0.962, and the prediction ability of the model was Q^2^ = 0.806. The closer these three indexes are to 1, the more stable and reliable the model is, and the fitting degree of the model is better. At the same time, the randomly repeated tests of *n* = 200 was adopted. As shown in Figure 2C,D, the R2 and Q2 values of the model of cucumber xylem and phloem sap on the far right exceeded 0.9, and the intercept of Q2 regression line was −0.943 and −1.09 respectively, which were both less than 0.05, indicating that the randomly repeated test was qualified, the model did not have over fitting phenomenon, and had good stability and predictability, it was suitable to explore the metabolite differences of cucumber xylem and phloem sap under different phosphorus levels.

### 2.4. Screening of Differential Metabolites

VIP > 1 based on PLS-DA model and *p* < 0.05 based on F test were used for screening, and the metabolites were shown in Table 1. A total of nine categories and 22 differential metabolites were selected from xylem sap under five phosphorus levels. The types of differential metabolites from more to less were organic acids, amino acids, sugars, alcohol amines, plant sterols and so on. A total of seven categories and 22 kinds of differential metabolites were selected from phloem sap. The types of differential metabolites from more to less are organic acids, amino acids, sugars, alcohol amines, phytosterols and so on.

As an important component of cucumber plants, the type and content of organic acids determine the flavor quality of cucumber [18]. Organic acids are mainly produced in mitochondria by the tricarboxylic acid cycle, stored in vacuoles [19]. The organic acids in the differential metabolites screened by this experiment mainly include stearic acid, palmitic acid, malic acid, citric acid, oxalic acid, succinic acid and so on. In the xylem and phloem differential metabolites of cucumber, organic acids were the most abundant, and their VIP values were all large at *p* < 0.05. Therefore, it is necessary to study organic acids.

As nitrogen-containing small molecular organic compounds, amino acids are essential nutrients for cucumber plant growth and development [20]. Amino acids are involved in nitrogen metabolism in cucumber plants and exist in the form of free states, polypeptides and proteins [21]. The amino acids in the differential metabolites screened out in this experiment mainly include phenylalanine, glutamic acid, γ-aminobutyric acid, etc. Amino acids are the second only to organic acids in the differential metabolites of cucumber xylem and phloem, which also have important significance.

Sugars are important nutrients and supporting substances in cucumbers, and play a pivotal role in the growth cycle of cucumbers [22]. Sugars in the differential metabolites screened in this experiment mainly include glucose and sucrose in phloem sap. Sugars not only provide energy for cucumber during its normal growth and development, but also have the function of signal transmission and are important regulatory factors of cucumber growth, development and gene expression. When regulating cucumber metabolism, sugars and other signals such as plant hormones form a complex signal network system [23].

### 2.5. Cluster Analysis of Differential Metabolites

**Figure 3:**
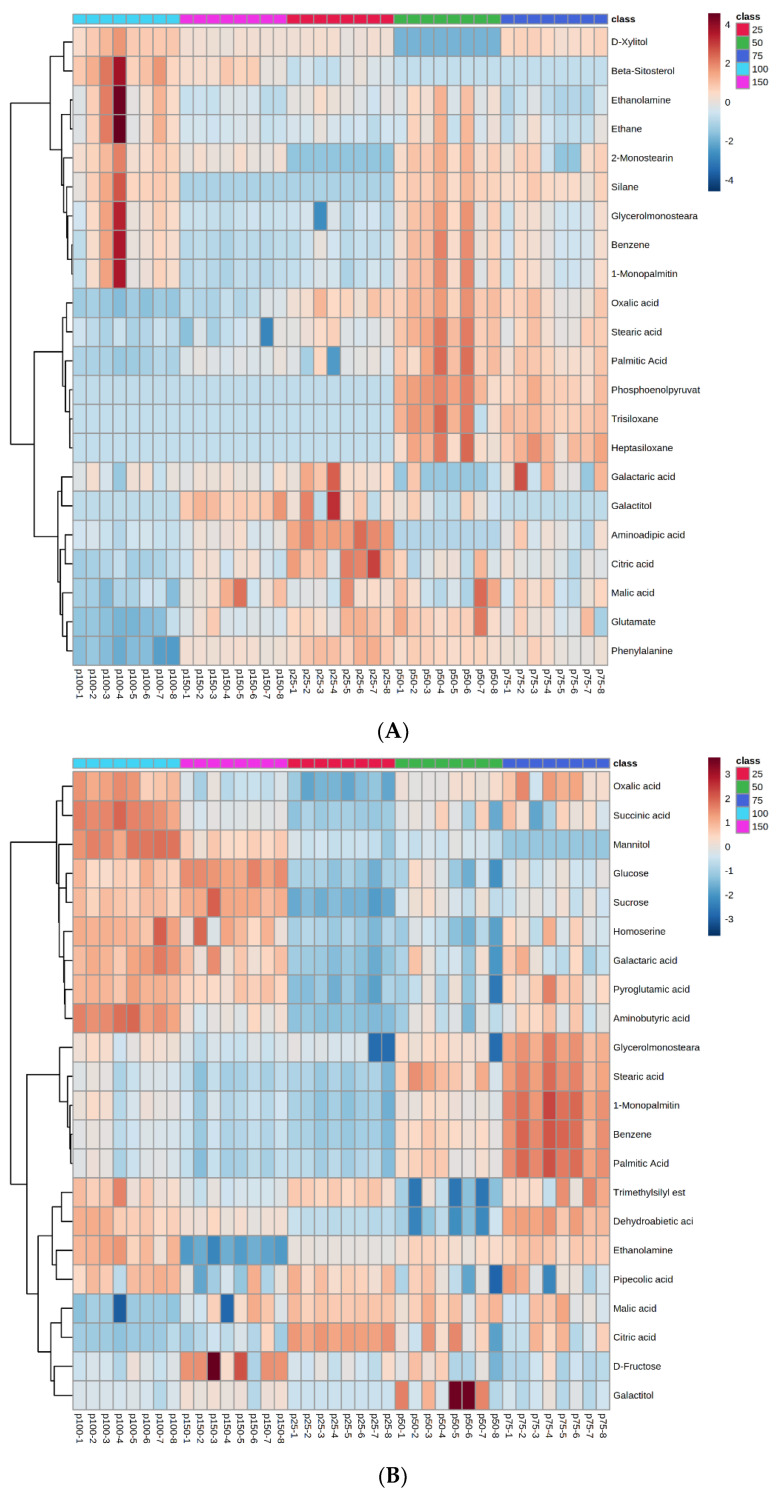
Correlation heat maps of differential metabolites between xylem and phloem sap of Cucumber under different phosphorus levels (xylem (**A**), phloem (**B**)).

Cluster analysis showed that different phosphorus levels had significant effects on the content of differential metabolites in cucumber xylem and phloem sap. The content change trend of differential metabolites in cucumber xylem and phloem sap under different phosphorus levels was expressed in the form of a heat map, as shown in Figure 3A,B. Each column in the figure represents a group of samples of cucumber xylem and phloem sap with different phosphorus levels, each row represents a differential metabolite, the color of the thermal diagram represents the content of differential metabolites in the group of samples, and the color from blue to white to red represents how the relative content of the differential metabolite gradually increases.

Figure 3A,B shows that the contents of differential metabolites of organic acids, mainly oxalic acid and citric acid in cucumber xylem sap and malic acid and citric acid in phloem sap, under low and high phosphorus stress were significantly higher than those under normal phosphorus supply. The contents of sugars, mainly sucrose and glucose, in differential metabolites were significantly increased under high phosphorus stress, and significantly decreased under low phosphorus stress. The contents of amino acids, mainly phenylalanine and glutamate, in xylem increased significantly under 25%, 50%, 75% and 150% phosphorus stress compared with normal phosphorus stress. The contents of amino acid mainly pyroglutamic acid and homoserine in phloem decreased significantly under 25%, 50%, 75% and 150% phosphorus stress compared with normal phosphorus stress.

### 2.6. Metabolic Pathway Analysis of Differential Metabolites

**Figure 4:**
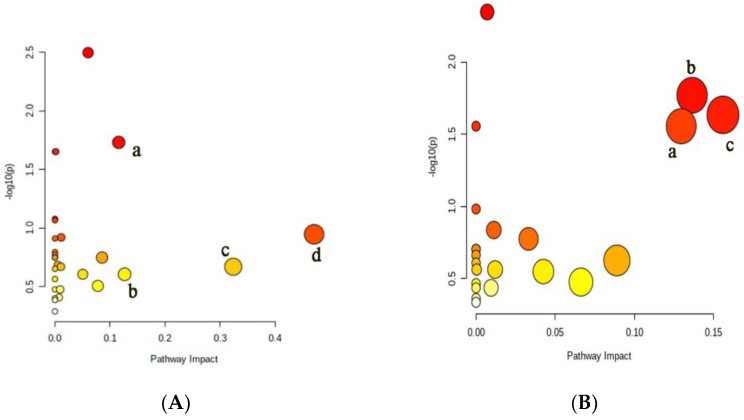
Analysis of metabolic pathways in xylem and phloem of cucumber. (**A**) Differential metabolite metabolic pathways in xylem: a. tricarboxylic acid cycle; b. glycolysis; c. alanine, aspartate and glutamate metabolism; d. phenylalanine metabolism; (**B**) differential metabolite metabolic pathways in phloem: a. metabolism of alanine, aspartate and glutamate; b. γ-aminobutyric acid metabolism; c. tricarboxylic acid cycle.

During the growth and development of cucumber plants, the metabolic activities are very complex and are jointly regulated by a variety of substances and reactions. They cannot be judged only by the content of some substances. Therefore, it is necessary to further analyze its metabolic pathway [24]. The metabolic pathways of differential small molecule metabolites in xylem and phloem sap of cucumber were analyzed, and the results were shown in Figure 4. The abscissa in the figure represents importance and the ordinate represents significance. The more right and upper the position is, the more differential the metabolic pathways are. There were four xylem metabolic pathways with Impact Value higher than 0.1, including the glycolysis, the alanine, aspartic acid and glutamate metabolism, the tricarboxylic acid cycle, and the phenylalanine metabolism. There were three phloem metabolic pathways with Impact Value higher than 0.1, including the tricarboxylic acid cycle, the alanine, aspartic acid and glutamic acid metabolism and the γ-aminobutyric acid metabolism.

**Figure 5:**
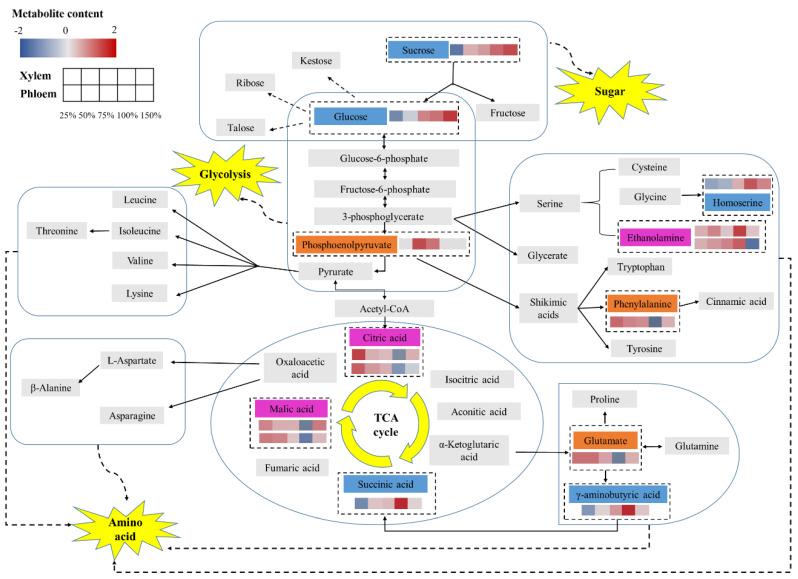
Metabolic network changes for xylem and phloem sap of cucumber upon phosphorus stress. The orange rectangle represents extremely significant differential metabolites on key pathways in xylem; the blue rectangle represents extremely significant differential metabolites on the key pathways in phloem; the purple rectangle represents extremely significant differential metabolites in key pathways shared by xylem and phloem; the first row (xylem) and the second row (phloem) had small hot boxes, showing the changes of key differentially expressed metabolites under phosphorus stress (P25%, P50%, P75%, P100%, P150%).

Through metabolic pathway analysis, the differential metabolites were mapped, which reflected there were extremely significant differential metabolites in xylem and phloem [25]. Among them, there were six species in xylem, including glutamate, phenylalanine, phosphoenolpyruvate, ethanolamine, malic acid and citric acid, and eight species in phloem, including γ-aminobutyric acid, homoserine, succinic acid, glucose, sucrose, ethanolamine, malic acid and citric acid. In order to understand the key metabolites more intuitively, the metabolic pathway network change diagram shown in Figure 5 was obtained, and the average value of the extremely significant differential metabolites content under the same phosphorus stress level in the thermodynamic diagram was extracted to make a small hot box [8]. Similarly, the color of the small hot box indicated the content of extremely significant differential metabolites in the group of samples, and the color from blue to white to red represented the gradual increase of the relative content of extremely significant differential metabolites.

Comprehensive analysis showed that the contents of glutamic acid and phenylalanine in xylem sap decreased firstly and then increased with the increase of phosphorus concentration, while the contents of γ-aminobutyric acid and homoserine in phloem sap increased firstly and then decreased with the increase of phosphorus concentration. The intermediate products of the tricarboxylic acid cycle showed a “first decrease and then increase” trend with the increase of phosphorus concentration. The succinic acid content in phloem sap increased firstly and then decreased with the increase of phosphorus concentration. Glucose and sucrose in phloem sap are upregulated during glucose metabolism. The phosphoenolpyruvate in xylem sap increased firstly and then decreased with the increase of phosphorus concentration. Ethanolamine in phloem showed a trend of “first decrease and then increase” with the increase of phosphorus concentration, while in xylem showed a trend of “increase–decrease–increase–decrease”.

## 3. Discussion

Existing studies have shown that plants are complex and delicate organisms, and the synthesis and decomposition of metabolites in vivo are always in a delicate dynamic equilibrium state to respond to changes in the external environment and maintain normal metabolism. [26]. Similarly, this research found that cucumber plants will also produce corresponding regulation strategies after phosphorus stress, involving amino acid metabolism, glycolysis metabolism, organic acid metabolism and other metabolic pathways.

Amino acids play an important role in plant response to stress. A large number of studies have confirmed that free amino acids in plants tend to accumulate under abiotic stress [27]. It can be seen from Figure 5 that glutamic acid content in cucumber xylem sap in this study showed a trend of “first decrease and then increase” with the increase of phosphorus concentration, indicating that glutamic acid content increased to alleviate the damage caused by stress conditions when phosphorus concentration was abnormal, which was consistent with the results of previous studies [28]. Shikimic acid produced by shikimic acid pathway generates phenylalanine through trans-ammonia reaction of branched acid and pre-phenylalanine, thus entering the phenylpropanoid metabolic pathway. Phenylalanine ammonia-lyase catalyzes ammonia-hydrolysis of L-phenylalanine to trans-cinnamic acid, which is an enzyme that connects primary metabolism with phenylpropanoid metabolism and catalyzes the first step reaction of phenylpropanoid metabolism. It is also a key enzyme and rate-limiting enzyme in the phenylalanine metabolic pathway [29,30,31]. The content of phenylalanine in cucumber xylem sap showed a trend of “first decrease and then increase” with the increase of phosphorus concentration. It may be because the activity of phenylalanine ammonia-lyase decreased under phosphorus stress, and the metabolic pathway of synthesizing cinnamic acid from phenylalanine was blocked, resulting in the accumulation of phenylalanine. γ-aminobutyric acid is a kind of non-protein amino acid containing four carbon atoms. As an important member of the free amino acid library [32], γ-aminobutyric acid also plays an important role in the regulation mechanism of plant metabolism. Previous studies have shown that γ-aminobutyric acid accumulates rapidly in plants under stress [27], but this study was inconsistent with the results of previous studies. Figure 5 showed that γ-aminobutyric acid content in phloem sap decreased under both low and high phosphorus stress compared with normal phosphorus level. Analysis suggested that it might be due to the blocked metabolic pathway of glutamate synthesis from α-ketoglutaric acid, thus affecting the process of glutamate synthesis from γ-aminobutyric acid [32]. Similar to γ-aminobutyric acid, the content of homoserine in phloem sap decreased under both low and high phosphorus stress to alleviate the damage caused by stress.

Sugar is not only a carbon source, energy and structural substance of cucumber plant cells, but also a signal molecule, which plays an important role in cucumber plant growth and development and response to stress [33]. Phosphorus stress can stimulate the changes of glycometabolism in cucumber plants. Previous studies have shown that the soluble sugar content of tomato, tobacco and other plants accumulated greatly under the condition of phosphorus deficiency [34,35], but the results of this study were different from those of previous studies. It can be seen from Figure 5 that the contents of glucose and fructose in the glycolysis pathway showed a positive correlation with the change of phosphorus concentration. Their contents under low phosphorus stress were lower than those under the normal phosphorus supply level, which limited effective glycolysis and might lead to carbon hunger and energy shortage [36]. However, the contents of glucose and fructose increased significantly under high phosphorus stress. It was speculated that there might be differences in the glycolytic cycle of different plants in response to the same stress, or it might be related to the different response mechanism of soluble sugar with different stress concentrations [37].

Studies have shown that many plants often make adaptive physiological responses to environmental stress through organic acid metabolism in vivo. Organic acids, especially some low molecular organic acids such as malic acid, citric acid and oxalic acid, participate in many physiological metabolic processes in plants [38]. This study also confirmed this phenomenon. As shown in Figure 5, phosphorus stress promoted the tricarboxylic acid cycle of cucumber xylem and phloem, resulting in the synthesis of a large number of organic acids. For example, the contents of malic acid and citric acid in cucumber xylem and phloem sap increased when the phosphorus concentration was too low or too high, indicating that in order to alleviate the damage caused by phosphorus stress, cucumber synthesized a large number of organic acids to maintain normal physiological and metabolic activities [8]. However, the content of succinic acid in phloem sap decreased at both low and high phosphorus levels, which may be caused by the decrease in the content of γ-aminobutyric acid that hindered the synthesis of succinic acid.

Through screening and metabolic pathway analysis of differential metabolites in cucumber xylem and phloem sap under five phosphorus levels, three common extremely significant difference metabolites were obtained from cucumber xylem and phloem sap, namely malic acid, citric acid and ethanolamine (Figure 5). Both malic acid and citric acid showed a “decrease first and then increase” trend with the increase of phosphorus concentration, while ethanolamine in phloem showed a “decrease first and then increase” trend with the increase of phosphorus concentration, and xylem showed an irregular pattern of “increase–decrease–increase–decrease”. Since ethanolamine in xylem and phloem sap varied with phosphorus concentration and the detection process was cumbersome, malic acid and citric acid responded to phosphorus stress in the same way, and the two were more sensitive to the change of phosphorus environment, that is, the content of malic acid and citric acid under low and high phosphorus stress was significantly higher than that under normal phosphorus supply. Therefore, it is preliminarily considered that malic acid and citric acid can be used as detection indexes to evaluate whether the phosphorus nutrient supply level of cucumber is normal under the ideal state when only phosphorus changed.

It should be pointed out that this research preliminarily conducted experimental analysis on the sap in phloem and xylem of “Jin yan 4” cucumber variety. The abundant compounds in phloem and xylem of different varieties of cucumber may have genetic variation. Moreover, the compounds in the sap of xylem and phloem of cucumber at different growth stages may also be different. These are worthy of further discussion and research. It is certain that the method of detecting metabolic biomarkers proposed in this research is also applicable to the detection of cucumber plants of different varieties and different growth stages. It can be used to analyze the effects of varieties, growth periods and other factors on compounds in phloem and xylem. Based on the method proposed in this research, we hope to establish a metabolic biomarker database by integrating the metabonomic data of phloem and xylem, so as to evaluate the phosphorus nutrient level of cucumber in different varieties and growth stages.

## 4. Materials and Methods

### 4.1. Experimental Instruments and Materials

The cultivar was cucumber cultivar “Jin yan 4”, and the experimental samples were cultivated in Venlo greenhouse of College of Agricultural Engineering, Jiangsu University. The experimental instruments and equipment used was: Gas chromatography mass spectrometer (GCMS-QP2010, Shimadzu Company, Tokyo, Japan); Capillary column (HP-5MS, 30 m × 0.25 mm × 0.25 μm, Agilent, Santa Clara, CA, USA); table-top high-speed refrigerated centrifuge (Neofuge 18R, Likang Biomedical Technology Holdings Co., Ltd., Shanghai, China); ultrasonic bathing apparatus (SK5200GT, Shanghai Kedao Company, Shanghai, China); Lyophilizer (FreeZone12Plus, Beijing Zhongkeker Instrument Co., Ltd., Beijing, China); electric blast drying oven (DH-101-2BY, Tianjin Zhonghuan Electric Furnace Co., Ltd., Tianjin, China).

### 4.2. Plant Sample Cultivation

Plant medium (Zhenjiang Peilei Matrix Technology Development Co., Ltd., Zhenjiang, China) was used for seedling cultivation, and the cucumber seedlings were transplanted when they had grown to three true leaves. In order to eliminate the influence of soil and other factors on the cultivation process of experimental samples, perlite cultivation mode was adopted after the samples were transplanted. On the premise of balance of other nutrient elements, phosphorus in nutrient solution was precisely regulated to obtain experimental samples under different phosphorus stress [39,40]. Yamasaki formula was used for nutrient solution, and the ion concentrations were 4.00 mmol·L^−1^ Ca^2+^, 1.50 mmol·L^−1^ Mg^2+^, 6.76 mmol·L^−1^ K^+^, 22.2 μmol·L^–1^ EDTA-Fe^2+^, 7.62 μmol·L^−1^ Mn^2+^, 0.76 μmol·L^−1^ Cu^2+^, 0.504 μmol·L^−1^ Zn^2+^, 1.50 mmol·L^–1^ SO_4_^2−^, 1.25 mmol·L^−1^ H_2_PO_4_^−^, 64.2 μmol·L^−1^ H_3_BO_3_, 0.495 μmol·L^−1^ H_2_MoO_4_, 15.26 mmol·L^−1^ NO_3_^−^, 0.5 mmol·L^−1^ NH_4_^−^. The nutrient solution was added at about 8:00 in the morning every day. When cucumber grows to seven leaves, 40 cucumber seedlings with basically the same growth status were selected (the deviation of plant height and stem diameter was less than 20%). They were divided into 4 experimental groups (32 plants, eight plants in each group) and control group (8 plants) and treated with five phosphorus gradient levels (25%, 50%, 75%, 100% and 150%). Among them, 100% phosphorus level was defined as the control group. The treatment of low phosphorus stress was to reduce H_2_PO_4_^−^ in nutrient solution to 0.3125, 0.625 and 0.9375 mmol·L^−1^ respectively; the treatment method of high phosphorus stress was to increase H_2_PO_4_^−^ in nutrient solution to 1.875 mmol·L^−1^. The greenhouse temperature ranged from 18–35 °C. The average daily irradiation was 13.2 MJ/m^2^, and the average natural light duration was 5.13 h. After two weeks, samples were taken between 10:00 and 12:00 am to minimize diurnal effects on metabolite concentration [5]. Qualitative and quantitative analysis was performed with GC-MS after the sampling was completed.

### 4.3. Sampling and Measurement

#### 4.3.1. Sampling of Xylem and Phloem Sap of Cucumber

Xylem and phloem were centrally distributed in the stem of cucumber plant. In this experiment, cucumber stem was selected to extract xylem and phloem sap. Because the main function of the xylem of cucumber is to transport the water and mineral nutrients absorbed by the root to various parts of the aboveground, it is generally considered to be bottom-up transport. The main function of cucumber phloem is to transport the photosynthetic products formed in leaves to all parts of the plant [41], which is generally considered to be top-down transport. Therefore, it is considered that the sap obtained from the incision near the ground end is xylem sap [42] and that from the other incision is phloem sap [43]. The cucumber stem 3–5 cm above the ground was cut off by using a sterilized sharp blade, the wound surface was cleaned with deionized water, then it was wiped with sterilized absorbent cotton, and samples were obtained at the end near the root and the sap was collected without taking the first drop. The xylem and phloem sap of eight cucumber plants in each of the five phosphorus level groups were collected with 2 mL sample tubes, and a total of 80 samples were obtained. All the collected samples were temporarily stored in liquid nitrogen for subsequent metabolite determination.

#### 4.3.2. GC-MS Metabolite Detection

Trimethylation derivatization treatment of samples: 30 µL of sap was taken from each sample, and then 170 µL of deionized water and 800 µL of methanol acetonitrile (1:1, *v*/*v*) were added to each sap. A frequency of 53 kHz was used to ultrasonicate the sample in an ice bath ultrasound for 1 h, and then the samples were incubated in a low-temperature refrigerator at −20 °C for 1 h to precipitate the protein. The samples were centrifuged in a high-speed refrigerated centrifuge at 12,000 r/min at 4 °C for 20 min, and 400 µL supernatant was collected. A total of 60 µL of ribitol (0.2 mg/mL) as an internal standard was added to each sample. The samples were oscillated in vortex oscillators for 10 s. Then, they were dried in a vacuum freeze dryer. A total of 80 µL (15 mg/mL) of methoxylamine pyridine solution was added and oscillated for 30 s. The samples were reacted at 37 °C for 90 min in a temperature-controlled incubator, and then 80 µL of BSTFA reagent (consisting of 1% TMCS) was added quickly. Finally, the samples were incubated in an incubator at 70 °C for 60 min.

Chromatographic conditions: the injection volume was 1 μL; The carrier gas was helium; the purging flow rate of the forward sample port was 3 mL/min; the flow rate was 1 mL/min; the initial column temperature was 90 °C and maintained for 15 s, after that, the column temperature was increased to 180 °C at the speed of 10 °C/min, then increased to 240 °C at the speed of 5 °C/min; finally, the column temperature was increased to 285 °C at the speed of 20 °C/min and kept for 11.5 min.

Mass spectrometry conditions: forward sample port temperature was 280 °C, transmission line temperature was 245 °C, ion source temperature was 220 °C. The ionization voltage was −70 eV. Scanning mode (full scanning): 20–600 m·z^−1^. The scanning speed was 100 spectra·s^−1^. The solvent delay was 480 s.

### 4.4. Data Analysis

The GCMS real-time analysis software of Shimadzu Instrument was used to extract peak area and retention time of each metabolite. Based on NIST database and Microsoft Excel, the peak areas of ribitol in each sample were used for qualitative and quantitative analysis of metabolites, and SIMCA 14.0 software was used for dimensionality reduction of experimental data. Both orthogonal partial least squares (OPLS-DA) and partial least squares (PLS-DA) models can be used for screening differential metabolites, but OPLS-DA is usually used for comparison between two groups, and PLS-DA can be used for classification comparison between two groups or more [44]. Therefore, the PLS-DA model was used to screen the differential metabolites of cucumber xylem and phloem sap under different phosphorus stress. Variables with weight of variation (VIP) value > 1 are generally considered to contribute more to differences, so their corresponding substances are considered as potential metabolites of differences. The substances with VIP > 1 were screened by PLS-DA model, and then non-parametric test was conducted with SPSS 22.0 software to further screen substances with *p* < 0.05. The substances with VIP > 1 and *p* < 0.05 were regarded as differential metabolites [45].

## 5. Conclusions

Based on GC-MS detection technology, combined with principal component analysis and partial least squares discriminant analysis, this research completed the preliminary screening of differential metabolites in cucumber xylem and phloem sap at five phosphorus levels and the correlation analysis of metabolic pathways. We systematically described the changes of the relative contents of differential metabolites under different phosphorus levels and the important metabolic pathways of cucumber xylem and phloem in response to phosphorus stress, providing abundant biochemical information for the metabolic response and regulation strategies of cucumber xylem and phloem under phosphorus stress. A total of 22 kinds of differential metabolites such as organic acids, amino acids, sugars and so on, were screened from cucumber xylem and phloem sap, respectively. Three common extremely significant differential metabolites were identified by metabolic pathway analysis of cucumber xylem and phloem sap, which were malic acid, citric acid and ethanolamine. Malic acid and citric acid in xylem and phloem sap made the same responses to phosphorus stress. Under low and high phosphorus stress, the contents of malic acid and citric acid were significantly higher than that under normal phosphorus supply level. It was considered that malic acid and citric acid could be used as the detection index to evaluate whether the phosphorus supply level of cucumber is normal under the ideal state when only phosphorus changed, but they could not be used as the marker of specific phosphorus level alone. In the future, further study will be carried out to explore the correlation between multiple factors, multiple metabolites and the phosphorus nutrient level of cucumber, so as to improve the accuracy of detection.

## Figures and Tables

**Table 1:** The differential metabolites in xylem and phloem sap of Cucumber under different phosphorus stress analyzed by PLS-DA model.

Xylem			Phloem		
Metabolites	*p*	VIP	Metabolites	*p*	VIP
Stearic acid	***	3.12029	Galactitol	***	3.33729
Palmitic acid	***	2.89369	Palmitic acid	***	3.12769
Phenylalanine	***	2.51584	Stearic acid	***	2.93922
Ethanolamine	***	2.39481	Galactaric acid	***	2.69905
Malic acid	***	2.15182	Pipecolic acid	***	2.64936
2-Monostearin	***	1.84387	Trimethylsilyl ester	***	2.44461
Beta-Sitosterol	***	1.67496	Ethanolamine	***	2.28112
Citric acid	***	1.61834	D-Fructose	***	2.15505
Silane	***	1.48680	γ-aminobutyric acid	***	1.76774
Glycerolmonostearate	***	1.45735	Oxalic acid	***	1.63812
Galactitol	***	1.44325	1-Monopalmitin	***	1.51261
Phosphoenolpyruvate	***	1.34310	Glycerolmonostearate	***	1.49872
1-Monopalmitin	***	1.31531	Glucose	***	1.46972
Glutamate	***	1.31233	Mannitol	***	1.27220
Oxalic acid	***	1.29589	Benzene	**	1.26719
Benzene	***	1.14935	Sucrose	***	1.15488
D-Xylitol	***	1.12737	Homoserine	***	1.15416
Trisiloxane	***	1.12260	Malic acid	**	1.15056
Galactaric acid	***	1.02495	Dehydroabietic acid	***	1.13394
Aminoadipic acid	***	1.00987	Succinic acid	**	1.11712
Heptasiloxane	***	1.00980	Citric acid	**	1.05460
Ethane	***	1.00030	Pyroglutamic acid	***	1.00308

Note: *** represents extremely significant (*p* < 0.001), and ** represents significant (*p* < 0.05).

## Data Availability

Data is contained within the article.
